# Chronic Q fever infective endocarditis: a case report

**DOI:** 10.1093/bjrcr/uaae017

**Published:** 2024-05-28

**Authors:** Badriya Al Suqri, Azza Al Brashdi

**Affiliations:** Nuclear Medicine Department, Royal Hospital, Muscat, PC: 111, Oman; Nuclear Medicine Department, Royal Hospital, Muscat, PC: 111, Oman

**Keywords:** Q fever, FDG PET/CT, Infective endocarditis, prosthetic valve

## Abstract

Q fever is an epidemic disease caused by the *Coxiella burnetii* infection. It can manifest clinically as an acute or chronic disease, with chronic infections being more common. Q fever endocarditis is the most common manifestation of chronic infection and usually occurs in patients with previous valvular heart disease like in our present study, a case of Q fever endocarditis that occurred in background of tetralogy of Fallot surgical repair. However, Q fever endocarditis is difficult to diagnose clinically and may lead to very serious or even life-threatening outcomes if not diagnosed promptly. In the present study, accurate diagnosis and treatment were achieved by 18F-FDG PET/CT combined with detection of the Q fever serological antibodies.

## Case presentation

We present a case of thirty-four-years-old male with a background of tetralogy of Fallot (TOF) surgicaly repaired in 1995, followed with surgical pulmonary valve replacement (Perimount 25) in 2012 for severe pulmonary regurgitation.

On October 30, 2018, he was admitted with bioprosthetic infective endocarditis (IE) (+ve blood culture + echo showed large vegetation on pulmonary bioprosthesis + CT chest: showed multiple septic emboli and mycotic aneurysm of right lower lobe pulmonary artery) which was treated medically. After recovery from IE, echocardiography showed no vegetation on the pulmonary valve but because of severe regurgitation, the patient underwent a successful percutaneous pulmonary valve implantation (PPVI) with 29-mm Edward SAPIEN Valve.

Echocardiography after PPVI showed the stent of PPVI in situ, mild paravalvular leak, and PG 24 mmHg.

## Investigations

The patient was lost for follow-up for 2 years after PPVI and admitted having been living in an area where he was taking care of cattle. When he presented for the OPD follow-up after 2 years, he has mild shortness of breath on exertion but denied any fever or other cardiac symptoms.

Investigations revealed high erythrocyte sedimentation rate (ESR92), and blood cultures were negative.

Transesophageal echocardiography showed Edward SAPIEN in 25-mm Perimount valve in pulmonary position with 2 mobile masses attached to valve probably vegetations, severe pulmonary valve regurgitation and pulmonary stenosis with a gradient of 40-50 mmHg along with mild-to-moderate tricuspid regurgitation (TR) with right ventricular systolic pressure (RVSP) of 60 mmHg.

Cardiac MRI was done and showed severe valvular and para valvular pulmonary regurgitation (PR) with right venticular end diastolic volume (RV EDV) of 174 ml.

The plan was for surgical pulmonary valve replacement after complete clearance from infection. Three sets of blood culture from 3 different sites were negative. Brucella antibodies and PCR were negative.

Q fever (*Coxiella burnetii*) showed phase I and phase II IgG at high levels with IGM compatible with a chronic infection.

## Differential diagnosis

The patient had 1 major criteria (findings in echocardiography) and only 1 minor diagnostic criteria (predisposing heart condition of prior prosthetic valve implantation) from the modified Duke criteria for IE, thus confirming a diagnosis of possible IE of the pulmonary valve prosthesis.

Because of the high likelihood of IE, 18F-FDG PET/CT was performed for further confirmation.

The patient was put on special preparation for cardiac PET procedures that include cardiac diet (high fat, very low/zero carbohydrates) for 72 h prior to performing the PET/CT scan and 6 h fast before the injection of 18F-FDG. He was then injected with a dose of 0.15 mCi/kg 18F-FDG and the PET/CT scan was performed from skull base to midthigh after approximately 60 min uptake period.

The ULTRA HD-PET (Tru + TOF) reconstruction method was used with scatter correction (RELATIVE) and GAUSSIAN filter.

**Figure 1.**
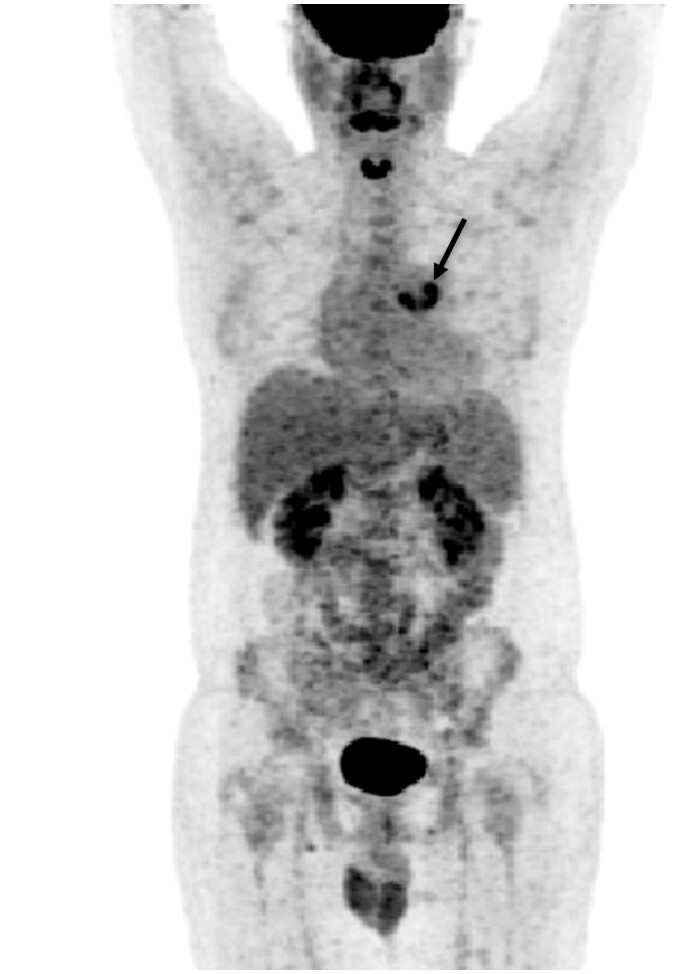
MIP image from 18FDG PET/CT scan showing intense focal uptake in the prosthetic valve (black arrow) in keeping with infective endocarditis.

**Figure 2.**
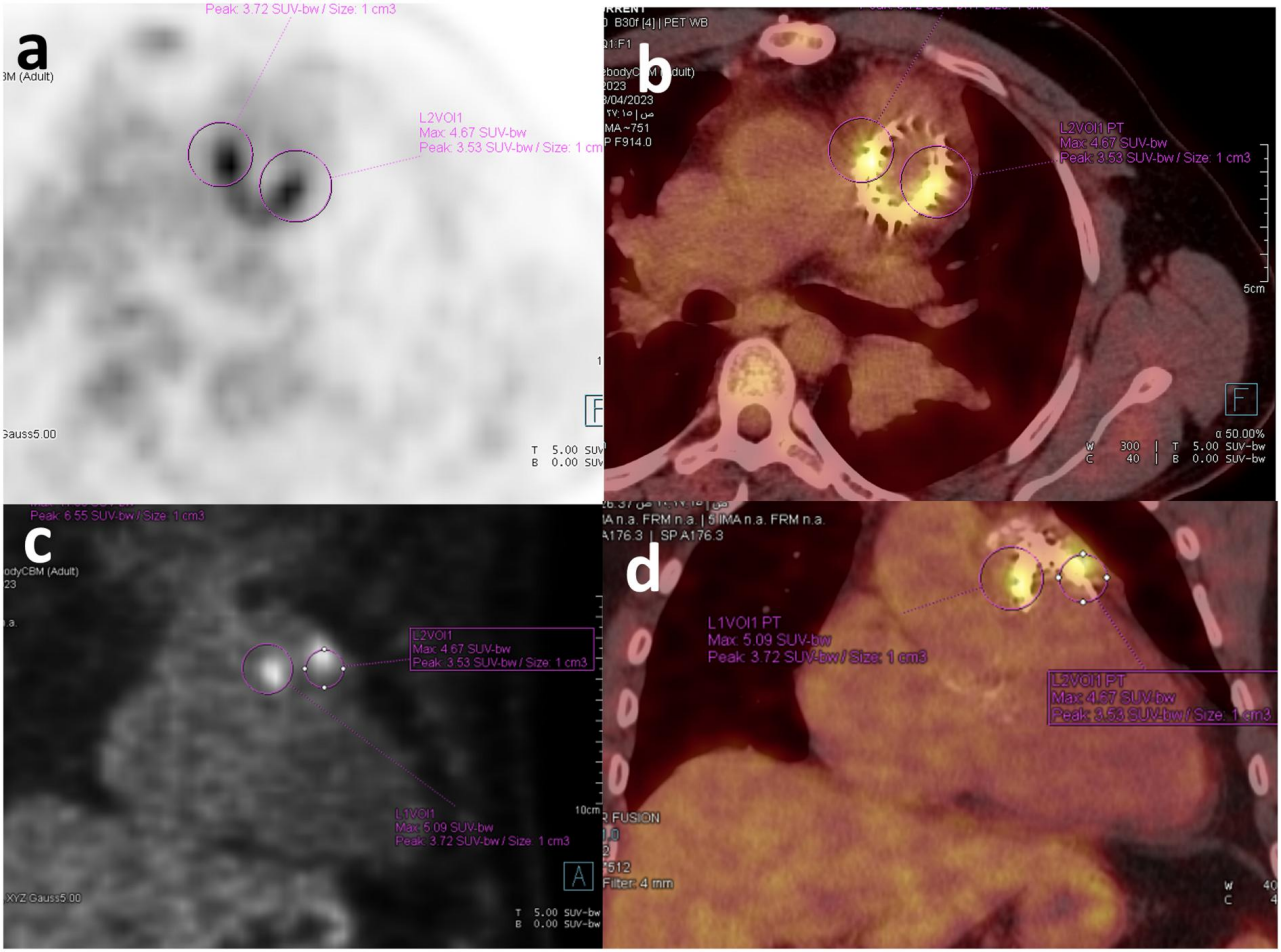
Axial (A) and coronal (C) PET images show two intense foci of increased FDG uptake seen to correspond to the prosthetic valve on the fused images (B and D).

**Figure 3.**
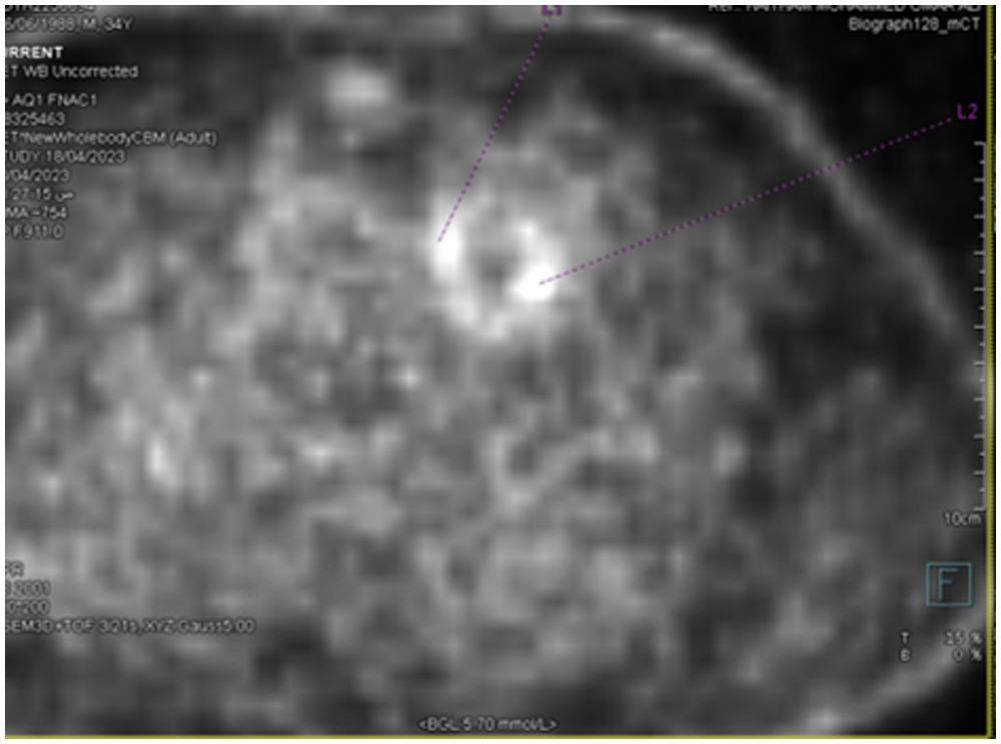
Axial non corrected FDG PET CT image.

The PET/CT scan showed focal increased FDG activity in the lateral and posterior aspects of the prosthetic pulmonary valve, SUV max up to 5 (Figures 1and 2). No other areas of abnormal FDG uptake were noted in the heart. Similar findings were noted in the non-attenuated (uncorrected) PET scan (Figure 3).

## Treatment

The patient was started on Q fever IE treatment: doxycycline 100 mg twice daily (bid) and hydroxychloroquine 200 mg three times a day. He subsequently underwent redo Pulmonary valve replacement (27-mm Avalus Bioprosthetic valve).

**Figure 4.**
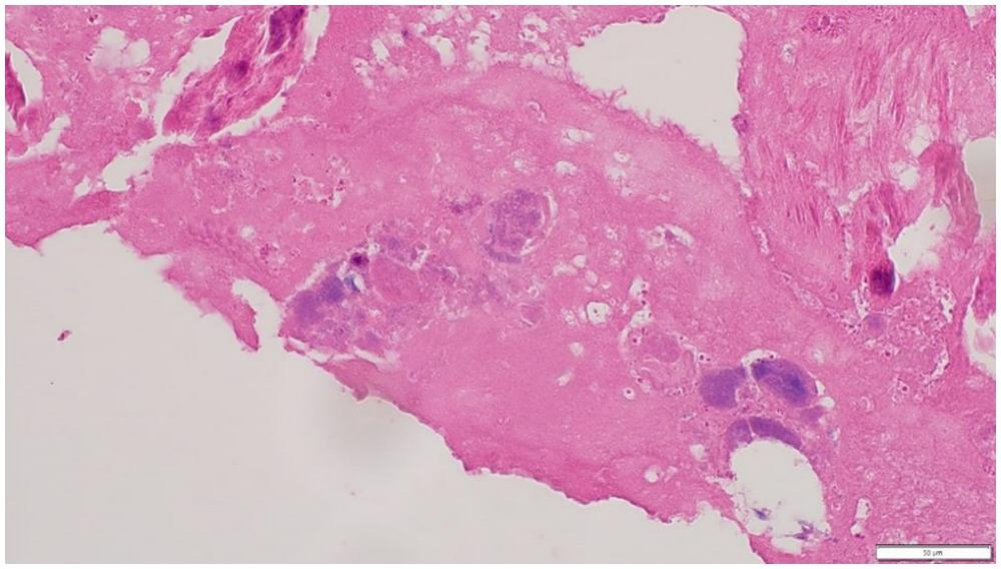
Hematoxylin and Eosin staining; magnification, ×40. Fibrin and bacterial colonies.

**Figure 5.**
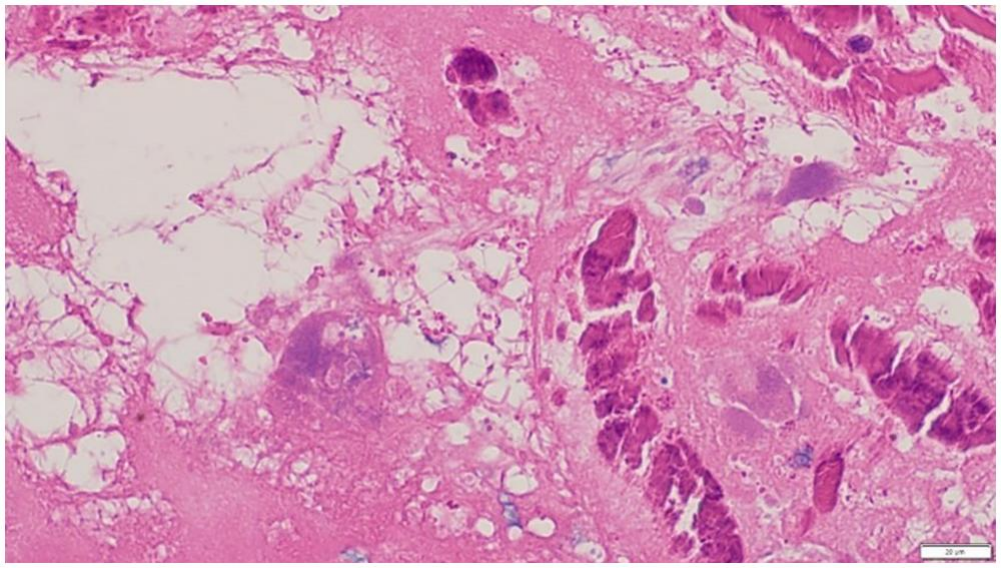
Hematoxylin and Eosin staining; magnification, ×40. Calcification with bacterial colonies.

**Figure 6.**
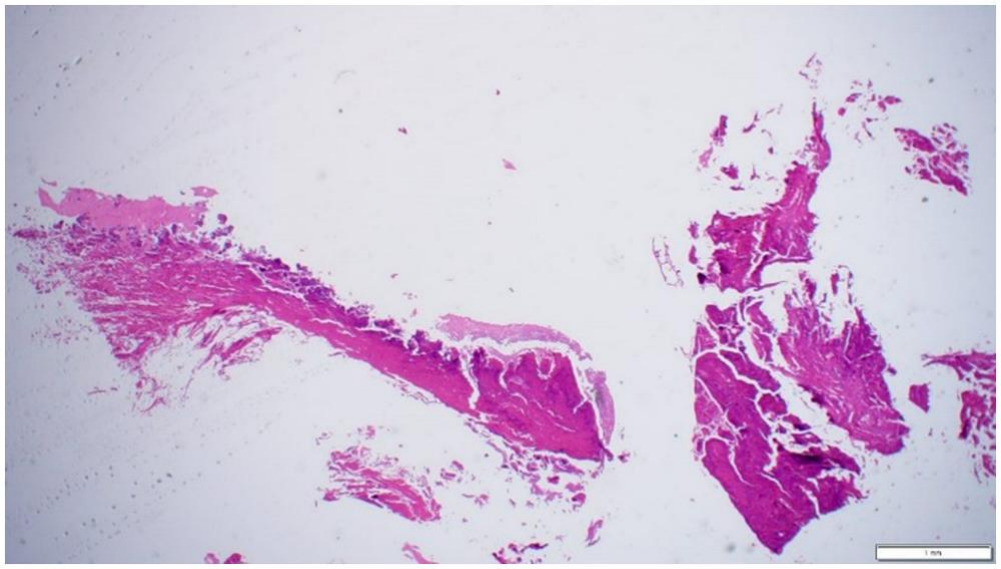
Hematoxylin and Eosin staining; magnification, ×40. Calcifications.

The excised pulmonary valve tissue microscopic examination revealed bacterial and fibrin colonies with extensive calcification are shown in (Figures 4 and 5). Zones of fibrous tissue (Figure 6) were found, which is suggestive of vegetations. Special statins were negative for microorganisms.

Q fever PCR came as positive.

## Outcome and follow-up

On further follow-up, the patient is asymptomatic and inflammatory markers are back to normal. Postsurgical Echocardiography revealed no more vegetations or intracardiac masses. The bioprosthetic valve seen in place with a presurre gradient (PG) of  17 mmHg. There was trace valvular leak and no paravalvular leak.

## Discussion

IE is a life-threatening disease with substantial morbidity and mortality. The incidence of the disease is increasing worldwide, likely due to an expanding option for cardiac valve repair and/or replacement and increasing use of cardiac-implanted devices.^1–3^

IE is a difficult-to-diagnose condition because of its highly variable clinical presentation. The diagnosis of this disease is based on the Duke criteria introduced in 1994 and modified in 2000,^4^ but their specificity and sensitivity are limited in cases of prosthetic valve endocarditis (PVE) and cardiac device-related infective endocarditis; hence, they should support rather than replace clinical judgement.^5^^,^^6^

In the case of PVE, FDG PET/CT has both high sensitivity and specificity for intracardiac infection. Laurens al. reported that FDG PET/CT had a sensitivity/specificity/positive predictive value/negative predictive value for PVE of 91%/95%/95%/91%, respectively.^7^ The most recent meta-analysis of this indication found similar data for PVE, but with low sensitivity (36%) and high specificity (99%) in native valve endocarditis.^8^^,^^9^

Blood culture-negative IE is a potentially severe disease that can be associated with infectious agents such as *Bartonella* spp., *C burnetii*, *Tropheryma whipplei*, and some fungi. The disease that is caused by *C burnetii* is Q fever. Q fever is a zoonotic disease which can either present in an acute or chronic form. Chronic Q fever mainly affects the heart where the disease manifests as endocarditis in 60%-80% of all cases of chronic Q fever worldwide.^10^

The diagnosis of Q fever endocarditis remains challenging. In a recent series, only 18% of cases showed the typical histological features of endocarditis, specifically, vegetations, inflammatory infiltrates and significant tissue destruction.^11^ More commonly, only extensive fibrosis and calcification were evident, which were difficult to discern from degenerative changes. *C burnetii* itself is not visible within macrophages on routine haematoxylin and eosin staining, and even special staining is unlikely to reveal the organism. Immunohistochemistry and polymerase chain reaction are more successful in demonstrating the presence of the organism; however, polymerase chain reaction has a sensitivity of only 75%.^11^

The optimum treatment of Q fever endocarditis includes doxycycline (100 mg/bid) and hydroxychloroquine (200 mg/bid) for the duration of 24 months. Furthermore, it is recommended to establish a goal of therapy of phase IgG titres less than 1:800 by maintaining doxycycline serum concentration 5 g/ml.^12^ Surgical replacement of the injured valve is often necessary for patients with Q fever endocarditis due to hemodynamic reasons.

## Learning points

- IE is a life-threatening disease with significant morbidity and mortality.
- Physicians should maintain a high index of suspicion of Q fever IE, especially among patients with pre-existing structural heart disease and associated risk factors.
- 18F FDG PET/CT plays an important role in confirming the diagnosis leading to well informed management plan.
- The diagnosis of IE due to chronic Q fever infection remains challenging, due to its highly variable clinical presentation. FDG PET/CT scan aids the diagnosis in patients with high clinical suspicion but inconclusive investigations.

## References

[1] Jensen AD , Bundgaard H, Butt JH, et al Temporal changes in the incidence of infective endocarditis in Denmark 1997-2017: a nationwide study. Int J Cardiol. 2021;326:145-152. 33069786 10.1016/j.ijcard.2020.10.029

[2] Heredia-Rodríguez M , Hernández A, Bustamante-Munguira J, et al Evolution of the incidence, mortality, and cost of infective endocarditis in Spain between 1997 and 2014. J Gen Intern Med. 2018;33(10):1610-1613. 29869145 10.1007/s11606-018-4514-7 PMC6153223

[3] van den Brink FS , Swaans MJ, Hoogendijk MG, et al Increased incidence of infective endocarditis after the 2009 European Society of Cardiology guideline update: a nationwide study in the Netherlands. Eur Heart J Qual Care Clin Outcomes. 2017;3(2):141-147. 28927175 10.1093/ehjqcco/qcw039

[4] Baddour LM , Wilson WR, Bayer AS, et al; American Heart Association Committee on Rheumatic Fever, Endocarditis, and Kawasaki Disease of the Council on Cardiovascular Disease in the Young, Council on Clinical Cardiology, Council on Cardiovascular Surgery and Anesthesia, and Stroke Council. Infective endocarditis in adults: diagnosis, antimicrobial therapy, and management of complications. Circulation. 2015;132(15):1435-1486. 26373316 10.1161/CIR.0000000000000296

[5] Prendergast BD. Diagnostic criteria and problems in infective endocarditis. Heart. 2004;90(6):611-613. 15145855 10.1136/hrt.2003.029850 PMC1768277

[6] Habib G , Lancellotti P, Antunes MJ; ESC Scientific Document Group. 2015 ESC Guidelines for the management of infective endocarditis: The Task Force for the Management of Infective Endocarditis of the European Society of Cardiology (ESC). Eur Heart J. 2015;36(44):3075-3128. 26320109 10.1093/eurheartj/ehv319

[7] Swart LE , Gomes A, Scholtens AM, et al Improving the diagnostic performance of 18F-fluorodeoxyglucose positron-emission tomography/computed tomography in prosthetic heart valve endocarditis. Circulation. 2018;138(14):1412-1427. 30018167 10.1161/CIRCULATIONAHA.118.035032

[8] Wang TK , Sanchez-Nadales A, Igbinomwanhia E, et al Diagnosis of infective endocarditis by subtype using 18F-fluorodeoxyglucose positron emission tomography/computed tomography: a contemporary meta-analysis. Circ Cardiovasc Imaging. 2020;13(6):e010600. 32507019 10.1161/CIRCIMAGING.120.010600

[9] Mahmood M , Kendi AT, Ajmal S, et al Meta-analysis of 18F-FDG PET/CT in the diagnosis of infective endocarditis. J Nucl Cardiol. 2019;26:922-935. 29086386 10.1007/s12350-017-1092-8

[10] Million M , Thuny F, Richet H, Raoult D. Long-term outcome of Q fever endocarditis: a 26-year personal survey. Lancet Infect Dis. 2010;10(8):527-535. 20637694 10.1016/S1473-3099(10)70135-3

[11] Lepidi H , Houpikian P, Liang Z, Raoult D. Cardiac valves in patients with Q fever endocarditis: microbiological, molecular, and histologic studies. J Infect Dis. 2003;187(7):1097-1106. 12660924 10.1086/368219

[12] Marrie TJ , Didier R. *Coxiella burnetii* (Q fever). In: Mandell GL, Bennett JE, Dolin R, eds. Mandell, Douglas, and Bennett’s Principles and Practice of Infectious Diseases. 7th ed. London: Churchill Livingstone/Elsevier; 2010:2511-2519.

